# COLUMBIA-1: a randomised study of durvalumab plus oleclumab in combination with chemotherapy and bevacizumab in metastatic microsatellite-stable colorectal cancer

**DOI:** 10.1038/s41416-024-02796-3

**Published:** 2024-07-25

**Authors:** Neil H. Segal, Jeanne Tie, Scott Kopetz, Michel Ducreux, Eric Chen, Rodrigo Dienstmann, Antoine Hollebecque, Matthew J. Reilley, Elena Elez, Jan Cosaert, Jason Cain, Yee Soo-Hoo, Nicola Hewson, Zachary A. Cooper, Rakesh Kumar, Josep Tabernero

**Affiliations:** 1 Memorial Sloan Kettering Cancer Center, New York, NY USA; 2 Weill Cornell Medical College, New York, NY USA; 3 Peter MacCallum Cancer Centre, Melbourne, VIC Australia; 4 MD Anderson Cancer Center, Houston, TX USA; 5 Paris-Saclay University, Gustave Roussy Cancer Center, Villejuif, France; 6 Princess Margaret Cancer Centre, Toronto, ON Canada; 7 Vall d’Hebron University Hospital and Institute of Oncology (VHIO), IOB-Quiron, Barcelona, Spain; 8 University of Vic-Central, University of Catalonia (UVic-UCC), Vic, Spain; 9 Oncoclínicas Precision Medicine, Oncoclínicas, São Paulo, Brazil; 10 Gustave Roussy Cancer Center, Villejuif, France; 11 University of Virginia Comprehensive Cancer Center, Charlottesville, VA USA; 12 AstraZeneca, Cambridge, UK; 13 AstraZeneca, Gaithersburg, MD USA

**Keywords:** Colon cancer, Tumour biomarkers, Cancer immunotherapy

## Abstract

**Background:**

To determine whether the addition of durvalumab (anti-PD-L1) and oleclumab (anti-CD73) to standard-of-care treatment (FOLFOX and bevacizumab) enhances the anti-tumour effect in patients with metastatic colorectal cancer (mCRC).

**Methods:**

COLUMBIA-1 (NCT04068610) was a Phase Ib (feasibility; Part 1)/Phase II (randomised; Part 2) trial in patients with treatment-naïve microsatellite stable mCRC. Patients in Part 2 were randomised to receive standard-of-care (control arm) or standard-of-care plus durvalumab and oleclumab (experimental arm). Primary objectives included safety and efficacy.

**Results:**

Seven patients were enrolled in Part 1 and 52 in Part 2 (*n* = 26 in each arm). Grade ≥3 treatment-emergent adverse events (TEAE) occurred in 80.8% and 65.4% of patients in the control and experimental arms of Part 2, respectively, with 26.9% and 46.3% experiencing serious TEAEs. The confirmed objective response rate (ORR) was numerically higher in the experimental arm compared with the control arm (61.5% [95% confidence interval (CI), 40.6–79.8] vs 46.2% [95% CI, 26.6–66.6]) but did not meet the statistically significant threshold in either arm.

**Conclusion:**

The safety profile of FOLFOX and bevacizumab in combination with durvalumab and oleclumab was manageable; however, the efficacy results do not warrant further development of this combination in patients with microsatellite stable mCRC.

**Registration:**

NCT04068610.

## Background

Despite advances in the treatment of metastatic colorectal cancer (mCRC), most patients still progress within a year of receiving first-line treatment with combination chemotherapy plus a biological agent (i.e., bevacizumab or cetuximab) [1]. Combination chemotherapy in this setting typically comprises 5-fluorouracil with folinic acid and either irinotecan (FOLFIRI), oxaliplatin (FOLFOX), or irinotecan and oxaliplatin (FOLFOXIRI); or alternatively, capecitabine with folinic acid and oxaliplatin (CAPOX) or irinotecan (CAPIRI) [1–5]. Immune checkpoint inhibition has shown remarkable clinical activity in a subset of ~4–5% of patients with mCRC whose tumours harbour microsatellite instability (MSI), a phenotype arising from deficient DNA mismatch repair mechanisms [6–9]. In the broader CRC population, including microsatellite-stable (MSS)-CRC, monotherapy with checkpoint inhibitors such as anti-programmed cell death-1 (PD-1) or anti-programmed cell death ligand-1 (PD-L1) antibodies has resulted in limited or no anti-tumour activity [8, 10].

Durvalumab is a human immunoglobulin G1 (IgG1) monoclonal antibody (mAb) that selectively blocks the interaction of PD-L1 with PD-1 on T cells and cluster of differentiation (CD) 80 on immune cells, and is engineered to reduce antibody-dependent cell-mediated cytotoxicity [11]. Data from a randomised Phase II clinical study demonstrated that durvalumab in combination with tremelimumab, an anti-cytotoxic T-lymphocyte-associated protein 4 (CTLA-4) antibody, improved overall survival (OS) in patients with MSS-mCRC compared with best supportive care [12]. More recently, the Phase Ib part of the CAMILLA trial reported tumour shrinkage in 4 of 17 (23.5%) patients with MSS-mCRC treated with durvalumab in combination with the multi-tyrosine kinase inhibitor, cabozantinib [13].

Adenosine is an immunosuppressive autocrine and paracrine factor that accumulates in the tumour microenvironment [14, 15]. Upon apoptotic or necrotic cell death, tumour cells release adenosine triphosphate (ATP) into the extracellular space, resulting in a pro-inflammatory response [15, 16]. CD39 and CD73 nucleotidases catalyse ATP to adenosine monophosphate (AMP) and AMP to immunosuppressive adenosine, respectively [17, 18]. Overexpression of CD73 has been associated with poor prognosis in multiple cancer types [18–20]. In patients with mCRC, overexpression of CD73 is an independent prognostic biomarker for survival [21, 22]. Combining a PD-L1 inhibitor such as durvalumab with a CD73 inhibitor may have a synergistic effect on reversing immune suppression in the tumour microenvironment. Indeed, preclinical studies have shown that treatment with anti-CD73 antibodies in combination with PD-1/PD-L1 inhibitors increased tumour inhibition and survival in tumour-bearing mice [19]. By introducing standard systemic chemotherapy to the PD-L1- and CD73-inhibitor combination, the anti-tumour effect may be further enhanced.

Oleclumab is a human IgG1 mAb that selectively inhibits CD73 and has shown potential for durable response in patients with MSS-mCRC [19, 23, 24]. In a Phase I first-in-human study investigating oleclumab with or without durvalumab in patients with advanced solid tumours (NCT02503774), one of 42 patients with MSS-mCRC had a complete response (CR) lasting 36.2 months and nine patients had stable disease (SD) [23]. In addition, a sustained decrease in CD73 level and enzymatic activity was observed in 37 samples of tumour cells, accompanied by an increase in 41 samples of CD8 + T cells [23]. Promising results were also seen with the combination of durvalumab and oleclumab in the randomised Phase II COAST trial (NCT03822351) in patients with unresectable Stage III non-small-cell lung cancer; the confirmed objective response rate (ORR) was numerically higher with durvalumab plus oleclumab (30.0%; 95% confidence interval [CI], 18.8–43.2) versus durvalumab alone (17.9%; 95% CI, 9.6–29.2), and the combination improved progression-free survival (PFS; hazard ratio, 0.44; 95% CI, 0.26–0.75) and the 12-month PFS rate (62.6% vs 33.9%) [25]. Thus, the combination of oleclumab with durvalumab is hypothesised to exert a synergistic effect to reverse immune suppression in the tumour microenvironment. In addition, chemotherapeutic agents, including oxaliplatin, are known to induce ATP release from tumour cells [26]; oleclumab may therefore counteract any downstream adenosine-mediated immunosuppressive effects induced by ATP release.

Here we report the results from COLUMBIA-1 (NCT04068610), a Phase Ib/II, open-label, multicentre study in treatment-naïve patients with MSS-mCRC that evaluated the safety and efficacy of combining FOLFOX and bevacizumab therapy with durvalumab and oleclumab.

## Methods

### Patients

Eligible patients were adults (age ≥18 years) with adequate organ function, an Eastern Cooperative Oncology Group performance status of 0 or 1, and histologically confirmed advanced or metastatic CRC. Patients were required to have had a documented mutation test and confirmed tumour location, and ≥1 measurable lesion by Response Evaluation Criteria in Solid Tumours (RECIST) v1.1 [27]; and must not have had defective DNA mismatch repair or MSI-high tumours. Additionally, patients should not have received prior treatment for recurrent or metastatic disease, with the exception of prior adjuvant chemotherapy or chemoradiotherapy if progression was >6 months after completing the adjuvant regimen. Patients were not eligible if they had received previous treatment with immunotherapy (i.e., CTLA-4 inhibitors or PD-1/PD-L1 inhibitors), anti-angiogenics (i.e., vascular endothelial growth factor [VEGF] or VEGF receptor inhibitors), or agents targeting CD73, CD39, or adenosine receptors. Patients were also excluded from the study if they had a history of venous thrombosis ≤3 months prior to the scheduled first dose of study treatment, or significant history of bleeding events or gastrointestinal perforation. The full inclusion and exclusion criteria for the study are provided in Supplementary Table S1.

### Study design

COLUMBIA-1 (NCT04068610) was a Phase Ib/II, open-label, multicentre, randomised, multi-drug platform study in treatment-naïve patients with MSS-mCRC that assessed the safety and efficacy of combining FOLFOX plus bevacizumab therapy with durvalumab and oleclumab using a two-part approach (Supplementary Fig. S1). The study recruited patients from Australia, Canada, France, Spain, and the USA from September 2019. Part 1 was a Phase Ib feasibility study with patients centrally assigned to FOLFOX (oxaliplatin 85 mg/m^2^ and folinic acid 400 mg/m^2^ intravenously [IV] every 2 weeks [q2w] on Day 1 [d1] of every cycle, and 5-fluorouracil 2400 mg/m^2^ IV over 46–48 h q2w on d1 and d2 of every cycle) plus bevacizumab (5 mg/kg IV q2w on d1) plus durvalumab (1500 mg IV q4w on d1) and oleclumab (3000 mg IV q2w on d1 for four doses, then q4w on d1). Part 2 was a randomised Phase II study that commenced upon confirmation of acceptable safety in Part 1. Patients were randomised to receive either FOLFOX plus bevacizumab (i.e., the control arm) or FOLFOX plus bevacizumab with durvalumab plus oleclumab (i.e., the experimental arm) at the same dosages as given in Part 1. A randomisation method with dynamically changing randomisation ratios was employed to account for fluctuation in the number of treatment arms open for randomisation over the course of the study. Study treatment continued until disease progression or any other discontinuation criteria were met. The current study used CONSORT reporting guidelines (Supplementary Table S2) [28].

### Study objectives

The primary objective in Part 1 was to evaluate safety by measuring incidence of adverse events (AEs), serious AEs, dose-limiting toxicities, and abnormal laboratory findings and vital signs; and in Part 2, to evaluate efficacy by determining the ORR per RECIST v1.1.

Secondary objectives in Part 1 and 2 included assessment of best overall response (BOR), duration of response (DoR), disease control rate (DCR), PFS per RECIST v1.1, 12-month PFS rate, and OS. Determination of ORR was a secondary objective for Part 1 only; safety was determined as a secondary endpoint for Part 2 only.

Exploratory objectives in Part 2 were to evaluate proteins that correlate with clinical activity of the experimental arm compared with the control arm through determining clinical activity in patients with varying CD73 and PD-L1 expression.

### Biomarker analyses

Tumour specimens were obtained during screening, haematoxylin- and eosin-stained slides were prepared centrally, and a pathologist examined a stained slide from each tissue block for the presence of viable tumour(s). From each patient sample, 4-μm thick sections were cut from a representative tumour block for immunohistochemistry (IHC) analysis. PD-L1 expression was tested using the Ventana antibody clone SP263 IHC assay on the Ventana Benchmark Auto-stainer (Roche Diagnostics, Ventana Medical Systems; Tucson, AZ, USA); PD-L1 was scored manually by a pathologist for percentage of tumour cells (TCs) with PD-L1 staining (i.e., TC%); tumour-infiltrating immune cell (IC) percentage (percentage of tumour area occupied by PD-L1 staining tumour-infiltrating ICs; IC%); and combined positive score (number of PD-L1 stained cells [TCs, lymphocytes, and macrophages]/total number of viable TCs × 100). CD73 IHC was performed with rabbit mAb clone D7F9A with a primary antibody concentration of 0.5 μg/mL (Cell Signaling Technology; Danvers, MA, USA) and scored manually by a pathologist for percent positive TCs with CD73 membrane expression at 1+/2+/3+ intensities. In addition, stromal CD73 expression was estimated as the percentage area of stroma staining at 1+/2+/3+ intensities. Tumours with ≥50% TCs positive for CD73 expression were classified as CD73 high; and <50% TCs positive for CD73 expression as CD73 low tumours. All IHC-stained slides were converted into high resolution digital images of the whole section (e-slide) using Aperio AT Turbo or Aperio XT scanners (Leica Biosystems; Buffalo Grove, IL, USA) with a ×20 objective magnification.

### Statistical analyses

The sample size calculation for the experimental arms was based on the primary endpoint, ORR. Assuming the ORR in the control arm is 55% [1] and the target ORR for the experimental arm is 75%, using the *χ*^2^-test, a total of 100 patients (i.e., 50 per arm) would provide ~80% power to have a statistically significant test outcome at the two-sided significance level of 0.2. The two-sided 95% CIs for ORR were estimated using the exact binomial distribution. The primary analysis compared the experimental arm with the control arm using the Cochran–Mantel–Haenszel (CMH) test stratified by the location of the primary tumour.

Analyses of secondary endpoints were performed as follows. The two-sided 95% CIs of each BOR rate and DCR was provided using an exact probability method. DCR was compared between the experimental arm and control arm using the CMH test stratified by the location of the primary tumour. DoR was analysed using the log-rank test for those patients with objective response in the as-treated population for Part 1 and the intent-to-treat population for Part 2. Median DoR with 95% CI was estimated using Kaplan–Meier method. PFS per RECIST v1.1 was analysed using the log-rank test stratified by the location of the primary tumour (left-sided vs right-sided). Median PFS and 12-month PFS rates with 95% CI were estimated using the Kaplan–Meier method. An estimate of the difference in 12-month PFS rates was performed under complementary log–log transformation of the Kaplan–Meier estimates of 12-month PFS rates [29]. OS was compared between the experimental arm and control arm using the log-rank test stratified by the location of the primary tumour. Median OS with 95% CI was estimated using the Kaplan–Meier method. However, the decision was made in February 2022 to terminate the clinical study early based on sponsor’s decision due to a lack of improvement in PFS and OS observed in the experimental arm compared with the control arm following interim analyses.

## Results

### Patient demographics and disease characteristics

As of 19 May 2023, 59 patients had been enrolled and received treatment; seven patients were included in Part 1 and 52 patients in Part 2 where 26 patients each were enrolled either in the control arm (FOLFOX + bevacizumab) or the experimental arm (FOLFOX + bevacizumab + durvalumab + oleclumab) (Supplementary Fig. S1). In total, 22 patients failed screening, due to failure to meet inclusion/exclusion criteria (*n* = 16), withdrawal of consent (*n* = 3), and other reasons (*n* = 3). Patient demographics and disease characteristics were broadly similar between the two parts, and between the control and experimental arms in Part 2 (Table 1). Median age was 57.0 (range, 45–79) and 58.5 (range, 22–80) years for patients in Part 1 and Part 2, respectively. In both parts of the trial, patients were predominantly male (57.1% and 65.4%, respectively), white (100% and 87.8%, respectively), and from the USA (85.7% and 63.5%, respectively). Disease histology indicated that 85.7% and 92.3% of patients in Part 1 and Part 2, respectively, were diagnosed with adenocarcinoma, while 14.3% and 3.8% were diagnosed with mucinous adenocarcinoma. Two patients had other histology in the experimental arm in Part 2 (Table 1). The most commonly reported sites of metastases were liver (71.4% in Part 1 and 90.4% in Part 2) and lung (57.1% and 46.2%). No metastases of the brain or central nervous system were reported.

**Table 1:** Patient demographics and disease characteristics.

	Part 1	Part 2		
	FOLFOX + bevacizumab + durvalumab + oleclumab (*n* = 7)	Total (*N* = 52)	Control arm: FOLFOX + bevacizumab (*n* = 26)	Experimental arm: FOLFOX + bevacizumab + durvalumab + oleclumab (*n* = 26)
Median age, years (range)	57.0 (45–79)	58.5 (22–80)	56.0 (22–72)	63.5 (41–80)
Male/female, *n* (%)	4 (57.1)/3 (42.9)	34 (65.4)/18 (34.6)	19 (73.1)/7 (26.9)	15 (57.7)/11 (42.3)
ECOG PS 0/1, *n* (%)^a^	5 (71.4)/2 (28.6)	30 (57.7)/17 (32.7)	12 (46.2)/12 (46.2)	18 (69.2)/5 (19.2)
Median weight, kg (range)/median BMI, kg/m^2^ (range)	81.6 (60.0–91.3)/24.4 (21.3–31.5)	77.9 (48.9–152.4)/27.2 (18.6–42.0)	76.7 (48.9–115.6) / 27.2 (18.6–36.6)	79.4 (57.1–152.4)/26.9 (19.8–42.0)
Race, *n* (%)^b^				
White	7 (100.0)	43 (87.8)	20 (87.0)	23 (88.5)
Asian	0	3 (6.1)	1 (4.3)	2 (7.7)
Black or African American	0	1 (2.0)	1 (4.3)	0
Other	0	2 (4.1)	1 (4.3)	1 (3.8)
Ethnicity, *n* (%)^c^				
Hispanic or Latino	0	5 (10.6)	4 (17.4)	1 (4.2)
Not Hispanic or Latino	7 (100.0)	42 (89.4)	19 (82.6)	23 (95.8)
Geographic region, *n* (%)				
USA	6 (85.7)	33 (63.5)	16 (61.5)	17 (65.4)
Spain	0	7 (13.5)	3 (11.5)	4 (15.4)
Australia	1 (14.3)	5 (9.6)	2 (7.7)	3 (11.5)
France	0	4 (7.7)	3 (11.5)	1 (3.8)
Canada	0	3 (5.8)	2 (7.7)	1 (3.8)
Sites of metastasis, *n* (%)^d^				
Liver	5 (71.4)	47 (90.4)	22 (84.6)	25 (96.2)
Lung	4 (57.1)	24 (46.2)	12 (46.2)	12 (46.2)
Histology, *n* (%)				
Adenocarcinoma	6 (85.7)	48 (92.3)	25 (96.2)	23 (88.5)
Mucinous adenocarcinoma	1 (14.3)	2 (3.8)	1 (3.8)	1 (3.8)
Other	0	2 (3.8)	0	2 (7.7)

*BMI* body mass index, *ECOG* Eastern Cooperative Oncology Group, *FOLFOX* folinic acid, 5-fluorouracil, and oxaliplatin, *PS* performance status.

^a^Data for two patients in the control arm and three patients in the experimental arm were not collected/missing.

^b^Each race category included only patients who selected only that category. Data for three patients in the control arm were not collected/missing.

^c^Data for three patients in the control arm and two patients in the experimental arm were not collected/missing.

^d^Part 1 included two (28.6%) instances of other visceral metastases; Part 2 included one (1.9%) instance of spleen metastasis in the control arm; Part 2 included seven (13.5%) instances of other visceral metastases (four [15.4%] in the control arm and three [11.5%] in the experimental arm).

### Safety

In Part 1, median exposure for all agents ranged from 43.9 to 56.3 weeks, except for oxaliplatin with a median exposure of 25.0 weeks (range, 6–33; Supplementary Table S3). Median exposure in Part 2 ranged from 32.5 to 34.3 weeks for all agents except for oxaliplatin with a median exposure of 22.2 weeks (range, 2–64; Supplementary Table S3). The median duration of exposure to all comparable agents was similar between patients in either treatment arms in Part 2.

All patients experienced treatment-emergent AEs (TEAEs), with 6/7 (85.7%) and 38/52 (73.1%) of patients having Grade ≥3 TEAEs in Part 1 and Part 2, respectively (Table 2). In Part 2, Grade ≥3 TEAEs occurred more often in the control arm (21/26 patients, 80.8%), than in the experimental arm (17/26 patients, 65.4%). The most commonly reported TEAEs in Part 2 were diarrhoea (50.0%), nausea (48.1%), and constipation, fatigue, and peripheral sensory neuropathy (40.4% each), with a similar incidence of these events in each arm (Table 3). Serious TEAEs were observed in 14.3% and 36.5% of patients in Part 1 and Part 2, respectively. In Part 2, serious TEAEs occurred less often in the control arm (26.9%) compared with the experimental arm (46.2%). A total of four deaths were reported due to TEAEs in the study, all of which were reported in Part 2. One death occurred in the control arm (COVID-19) and three deaths occurred in the experimental arm (due to intestinal perforation, peritonitis, and sepsis); none of these deaths were deemed to be related to any of the study treatments.

**Table 2:** Safety overview of TEAEs and TRAEs.

	Part 1	Part 2		
	FOLFOX + bevacizumab + durvalumab + oleclumab (*n* = 7)	Total (*N* = 52)	Control arm: FOLFOX + bevacizumab (*n* = 26)	Experimental arm: FOLFOX + bevacizumab + durvalumab + oleclumab (*n* = 26)
Any TEAE, *n* (%)	7 (100.0)	52 (100.0)	26 (100.0)	26 (100.0)
Grade ≥3	6 (85.7)	38 (73.1)	21 (80.8)	17 (65.4)
Serious TEAEs, *n* (%)	1 (14.3)	19 (36.5)	7 (26.9)	12 (46.2)
Death^a^, *n* (%)	0	4 (7.7)	1 (3.8)	3 (11.5)
TEAEs leading to discontinuation of:				
Durvalumab, *n* (%)	2 (28.6)	2 (3.8)	N/A	2 (7.7)
Oleclumab, *n* (%)	2 (28.6)	2 (3.8)	N/A	2 (7.7)
Durvalumab-related				
AEs, *n* (%)	4 (57.1)	19 (36.5)	N/A	19 (73.1)
SAEs, *n* (%)	1 (14.3)	1 (1.9)	N/A	1 (3.8)
Oleclumab-related				
AEs, *n* (%)	4 (57.1)	19 (36.5)	N/A	19 (73.1)
SAEs, *n* (%)	1 (14.3)	2 (3.8)	N/A	2 (7.7)

*AE* adverse event, *FOLFOX* folinic acid, 5-fluorouracil, and oxaliplatin, *N/A* not applicable, *SAE* serious adverse event, *TEAE* treatment-emergent adverse event, *TRAE* treatment-related adverse event.

^a^No deaths were found to be related to any treatment component.

**Table 3:** TEAEs occurring in >15% of total number of patients in Part 2.

	Part 1	Part 2		
	FOLFOX + bevacizumab + durvalumab + oleclumab (*n* = 7)	Total (*N* = 52)	Control arm: FOLFOX + bevacizumab (*n* = 26)	Experimental arm: FOLFOX + bevacizumab + durvalumab + oleclumab (*n* = 26)
Diarrhoea	5 (71.4)	26 (50.0)	12 (46.2)	14 (53.8)
Nausea	2 (28.6)	25 (48.1)	13 (50.0)	12 (46.2)
Constipation	3 (42.9)	21 (40.4)	9 (34.6)	12 (46.2)
Fatigue	5 (71.4)	21 (40.4)	10 (38.5)	11 (42.3)
Peripheral sensory neuropathy	5 (71.4)	21 (40.4)	11 (42.3)	10 (38.5)
Stomatitis	3 (42.9)	13 (25.0)	5 (19.2)	8 (30.8)
Decreased appetite	1 (14.3)	13 (25.0)	5 (19.2)	8 (30.8)
Dysgeusia	2 (28.6)	12 (23.1)	8 (30.8)	4 (15.4)
Paraesthesia	5 (71.4)	12 (23.1)	7 (26.9)	5 (19.2)
Vomiting	0	10 (19.2)	4 (15.4)	6 (23.1)
Temperature intolerance	1 (14.3)	10 (19.2)	5 (19.2)	5 (19.2)
Dehydration	2 (28.6)	9 (17.3)	3 (11.5)	6 (23.1)
Abdominal pain	1 (14.3)	8 (15.4)	5 (19.2)	3 (11.5)
Pyrexia	2 (28.6)	8 (15.4)	3 (11.5)	5 (19.2)
Neutrophil count decreased	4 (57.1)	8 (15.4)	4 (15.4)	4 (15.4)
Headache	1 (14.3)	8 (15.4)	5 (19.2)	3 (11.5)
Neuropathy peripheral	0	8 (15.4)	3 (11.5)	5 (19.2)
Dyspnoea	1 (14.3)	8 (15.4)	6 (23.1)	2 (7.7)
Epistaxis	0	8 (15.4)	5 (19.2)	3 (11.5)
Hypertension	3 (42.9)	8 (15.4)	4 (15.4)	4 (15.4)

*FOLFOX* folinic acid, 5-fluorouracil, and oxaliplatin, *TEAE* treatment-emergent adverse event.

Durvalumab treatment-related AEs (TRAE) occurred in four (57.1%) patients in Part 1 and 19 (73.1%) patients in the experimental arm of Part 2 (Table 2). Oleclumab TRAEs occurred in four (57.1%) patients in Part 1 and 19 (73.1%) patients in the experimental arm of Part 2 (Table 2). Seventeen (32.7%) patients in Part 2 had TRAEs considered to be related to both durvalumab and oleclumab. TRAEs occurring in ≥10% of total patients in Part 2 are summarised in Supplementary Table S4. A serious case of Grade 4 colitis (14.3%) in Part 1 was deemed to be related to both durvalumab and oleclumab. One case of Grade 1 pyrexia (3.8%) was deemed serious and related to durvalumab in the experimental arm of Part 2. Serious oleclumab-related TRAEs occurred in two (7.7%) patients in the experimental arm of Part 2: one case each of Grade 1 pyrexia and Grade 3 pulmonary embolism.

Treatment with both durvalumab and oleclumab was discontinued in two patients in Part 1 (one patient with Grade 4 large intestine perforation and one patient with Grade 2 pneumonitis); in Part 2 both drugs were discontinued in two patients, due to Grade 3 rectal abscess in one patient and Grade 3 pulmonary embolism in another.

### Efficacy

**Fig. 1:**
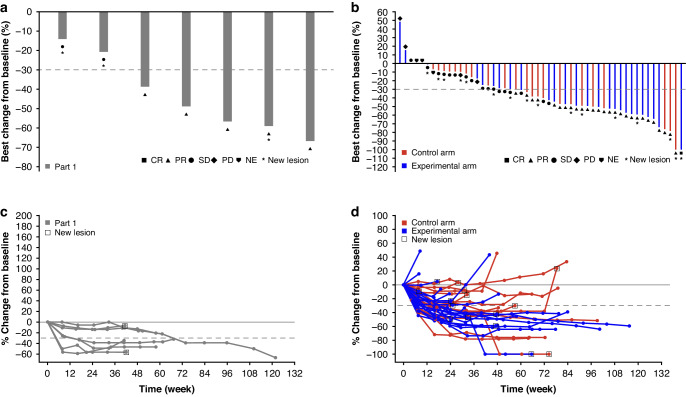
Anti-tumour response by best percentage change from baseline. Waterfall plots of anti-tumour response in (**a**) Part 1 and (**b**) Part 2; Spider plots of anti-tumour response in (**c**, **d**) Part 2. Part 1 and experimental arm: FOLFOX + bevacizumab + durvalumab + oleclumab. Control arm: FOLFOX + bevacizumab. CR complete response, FOLFOX folinic acid, 5-fluorouracil, and oxaliplatin, NE not evaluable, PD progressive disease, PR partial response, SD stable disease.

In Part 1, the confirmed ORR (i.e., CR + partial response [PR]) was 71.4% (95% CI, 29.0–96.3; Table 4, Fig. 1a). Median DoR in Part 1 was 6.0 months (95% CI, 5.5–not evaluable [NE]). In Part 2, the confirmed ORR was 61.5% (95% CI, 40.6–79.8) in the experimental arm (including one patient with a confirmed CR) compared with 46.2% (95% CI, 26.6–66.6) in the control arm (*P* = 0.317; odds ratio [OR], 1.8 [95% CI, 0.6–5.6]; Table 4 and Fig. 1b). The DCR (i.e., CR + PR + SD) was 100% (95% CI, 59.0–100) in Part 1. In Part 2, the DCR was 84.6% (95% CI, 65.1–95.6) and 88.5% (95% CI, 69.8–97.6) in the experimental and control arm respectively (*P* = 0.645; OR, 0.8 [95% CI, 0.2–4.3]). Median DoR for the experimental arm was 10.3 months (95% CI, 5.8–NE) compared with 7.7 months (95% CI, 4.6–15.4) in the control arm. Assessment of best changes in tumour size from baseline showed that responses were generally durable (Fig. 1c, d).

**Table 4:** Disease response and survival data in the ITT population.

	Part 1	Part 2		
	FOLFOX + bevacizumab + durvalumab + oleclumab (*n* = 7)	Total (*N* = 52)	Control arm: FOLFOX + bevacizumab (*n* = 26)	Experimental arm: FOLFOX + bevacizumab + durvalumab + oleclumab (*n* = 26)
Best overall response, *n* (%)^a^				
Confirmed CR^b^	0	1 (1.9)	0	1 (3.8)
Confirmed PR^b^	5 (71.4)	27 (51.9)	12 (46.2)	15 (57.7)
SD	2 (28.6)	17 (32.7)	11 (42.3)	6 (23.1)
Unconfirmed CR or PR	0	3 (5.8)	2 (7.7)	1 (3.8)
PD	0	4 (7.7)	1 (3.8)	3 (11.5)
NE	0	3 (5.8)	2 (7.7)	1 (3.8)
ORR, % (95% CI)^c^	71.4 (29.0–96.3)	53.8 (39.5–67.8)	46.2 (26.6–66.6)	61.5 (40.6–79.8)
DCR, % (95% CI)^d^	100 (59.0–100.0)	86.5 (74.2–94.4)	88.5 (69.8–97.6)	84.6 (65.1–95.6)
Median DoR, months (95% CI)	6.0 (5.5–NE)	10.3 (6.6–14.9)	7.7 (4.6–15.4)	10.3 (5.8–NE)
PFS				
Median PFS, months (95% CI)	9.5 (7.8–18.2)	10.9 (7.4–13.8)	11.1 (7.3–16.2)	10.9 (6.9–15.1)
12-month PFS rate, % (95% CI)	28.6 (4.1–61.2)	37.3 (23.2–51.4)	38.6 (18.6–58.3)	36.1 (17.1–55.5)
OS				
Median OS, months (95% CI)	30.1 (11.9–NE)	NR (18.3–NE)	NR (20.6–NE)	22.4 (10.6–NE)

*CI* confidence interval, *CR* complete response, *DCR* disease control rate, *DoR* duration of response, *FOLFOX* folinic acid, 5-fluorouracil, and oxaliplatin, *ITT* intention-to-treat, *NE* not evaluable, *NR* not reached, *ORR* overall response rate, *OS* overall survival, *PD* progressive disease, *PFS* progression-free survival, *PR* partial response, *RECIST* response evaluable criteria in solid tumours, *SD* stable disease.

^a^Based on application of RECIST v1.1 to investigator assessments.

^b^Defined as responses that were ongoing and confirmed after 4 weeks.

^c^Responses exclude unconfirmed responses.

^d^Defined as confirmed CR + confirmed PR + SD lasting 16 weeks.

**Fig. 2:**
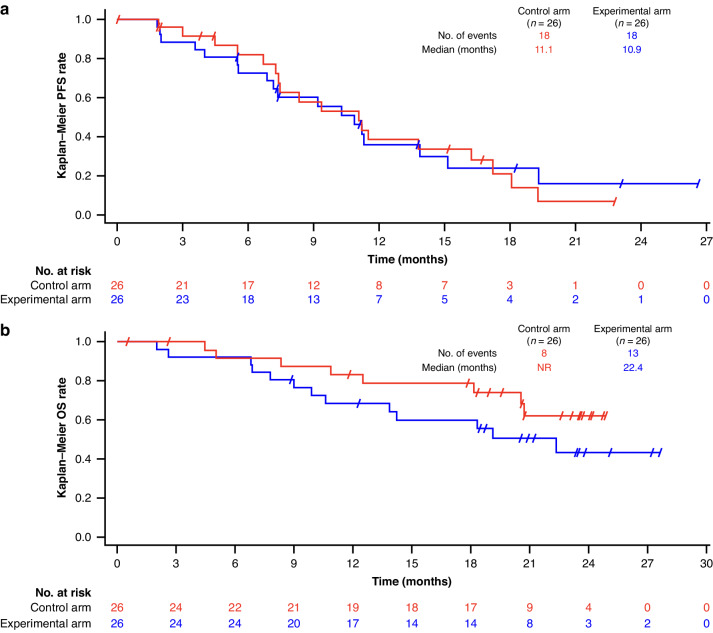
Survival data for Part 2. Kaplan-Meier curves indicacting (a) PFS and (b) OS. Control arm: FOLFOX + bevacizumab. Experimental arm: FOLFOX + bevacizumab + durvalumab + oleclumab. FOLFOX, folinic acid, 5-fluorouracil, and oxaliplatin; OS, overall survival; PFS, progression-free survival.

Median PFS for Part 1 was 9.5 months (95% CI, 7.8–18.2), with a 12-month PFS rate of 28.6% (95% CI, 4.1–61.2; Table 4); median OS was 30.1 months (95% CI, 11.9–NE). In Part 2, there were no significant differences in median PFS between the experimental and control arms, with a median PFS of 10.9 months (95% CI, 6.9–15.1) and 11.1 months (95% CI, 7.3–16.2), respectively (*P* = 0.588). The 12-month PFS rates were also similar, at 36.1% (95% CI, 17.1–55.5) and 38.6% (95% CI, 18.6–58.3), respectively (*P* = 0.866; Fig. 2a). In Part 2, median OS for the experimental arm was 22.4 months (95% CI, 10.6–NE), while it was not reached (95% CI, 20.6–NE) in the control arm (*P* = 0.216; Fig. 2b).

### Biomarkers

Forty-seven samples were evaluable for both PD-L1 and CD73 expression. The exploratory biomarker analyses showed that patients with CD73-high tumours (CD73-positive expression in ≥50% TCs) who received the experimental treatment had a numerically higher ORR of 72.7% (95% CI, 39–94) compared with those in the control arm (35.7% [95% CI, 12.8–64.9]). In patients with CD73-low tumours (CD73-positive expression in <50%), ORR was similar between the two arms: 58.3% [95% CI, 27.7–84.8] in the experimental arm and 60.0% [95% CI, 26.2–87.8] in the control arm (Supplementary Table S5). Additional analyses of best change in tumour size from baseline revealed a trend for an increased depth of response in patients with CD73 high tumours who received experimental treatment compared with the control regimen (Supplementary Fig. S2). Median PFS was numerically longer in patients with CD73-high tumours compared with those with CD73-low tumours, but was similar between the experimental and control arm in each subgroup (Supplementary Fig. S3). No clear observations were made for median OS by CD73 status for the experimental and control groups due to small sample sizes (Supplementary Table S5). No trends were observed between PD-L1 expression levels and efficacy outcomes with anti-PD-L1 therapy (data not shown).

## Discussion

This Phase Ib/II, open-label, multicentre, randomised study evaluated the safety and efficacy of FOLFOX and bevacizumab in combination with durvalumab and oleclumab in patients with treatment-naïve MSS-mCRC. The safety profile of FOLFOX and bevacizumab in combination with durvalumab and oleclumab was manageable with no new safety signals; the occurrences of diarrhoea, nausea, constipation, and fatigue reported in approximately 40–50% of patients in the experimental arm (FOLFOX and bevacizumab in combination with durvalumab and oleclumab) in Part 2 of the study are comparable to those described in prior studies combining anti-PD-L1 therapy with FOLFOX [30, 31]. Moreover, the combination of anti-PD-L1 therapy with anti-CD73 therapy has also previously demonstrated a manageable safety profile in patients with other advanced solid tumours [23, 25]. In the current study, however, a distinct pattern was noted of a higher incidence of Grade ≥3 TEAEs in the control arm of Part 2, in contrast to the experimental arm of Part 2 which had a higher incidence of serious TEAEs. Among 12 patients with serious TEAEs, only one was considered related to durvalumab and two related to oleclumab. Although four deaths resulting from TEAEs occurred in Part 2 of the study (one death due to COVID-19 in the control arm; one death each due to intestinal perforation, peritonitis, and sepsis in the experimental arm), no fatalities were found to be due to TRAEs.

In terms of anti-tumour activity, the addition of durvalumab and oleclumab to FOLFOX and bevacizumab showed a numerical increase in ORR (i.e., CR + PR) compared with FOLFOX and bevacizumab alone (61.5% vs 46.2%, respectively). However, the DCR (i.e., CR + PR + SD) was similar between the arms (84.6% vs 88.5%, respectively). Therefore, with almost double the patients experiencing SD in the control arm compared with the experimental arm (42.3% vs 23.1%), the moderate increase in response rates was not translated to improved PFS or OS as patients with SD likely remained in survival assessment.

Although patients were randomised in COLUMBIA-1, the lack of stratification according to baseline tumoral CD73 expression is a limitation of the study. Therefore, baseline demographics and disease characteristics, extent of disease at diagnosis, and any prior adjuvant chemotherapy or chemoradiotherapy may not be comparable between patients included in the two exploratory biomarker analysis groups. Comparison between the groups should also be considered within the limits of the small patient populations; and these efficacy analyses were conducted as exploratory ad hoc analyses from available and suitable data with different CD73 scoring methodologies from prior studies [21, 22]. Results from the exploratory biomarker analyses indicated moderate numerical increases in tumour response rates in the experimental versus control arm in patients with high tumoral CD73 expression, and longer PFS in both the experimental and control arms in patients with high CD73 expression compared with those with low CD73 expression. In the CD73-high group of patients, the numerically higher ORR seen in the experimental arm did not appear to translate into longer PFS and OS. Nevertheless, the improvement in PFS seen in patients with CD73-high tumour expression treated with FOLFOX and bevacizumab, with or without the addition of durvalumab and oleclumab, as compared to patients with CD73-low expression suggests the prognostic value of CD73. However, these results are in contrast with the documented poor prognosis associated with CD73 overexpression [21, 22], and further highlights the exploratory nature of our analysis and small sample size. Another limitation of this study may be the predominance of patients with liver metastases, with their presence known to impact the efficacy of immunotherapy in patients with MSS-mCRC [32].

In conclusion, the efficacy results from this trial do not support further development of the durvalumab/oleclumab/FOLFOX/bevacizumab combination in patients with MSS-mCRC. However, the observed prognostic value of CD73 tumour expression in MSS-mCRC and emerging data for durvalumab plus oleclumab in other tumour types do warrant further exploration.

## Supplementary information

Supplementary

## Data Availability

Data underlying the findings described in this manuscript may be obtained in accordance with AstraZeneca’s data sharing policy described at https://astrazenecagrouptrials.pharmacm.com/ST/Submission/Disclosure. Data for studies directly listed on Vivli can be requested through Vivli at www.vivli.org. Data for studies not listed on Vivli could be requested through Vivli at https://vivli.org/members/enquiries-about-studies-not-listed-on-the-vivli-platform/. The AstraZeneca Vivli member page is also available outlining further details: https://vivli.org/ourmember/astrazeneca/.

## References

[1] Venook AP, Niedzwiecki D, Lenz HJ, Innocenti F, Fruth B, Meyerhardt JA, et al. Effect of first-line chemotherapy combined with cetuximab or bevacizumab on overall survival in patients with KRAS wild-type advanced or metastatic colorectal cancer: a randomized clinical trial. J Am Med Assoc. 2017;317:2392–401. 10.1001/jama.2017.7105 PMC5545896 28632865

[2] Loupakis F, Cremolini C, Masi G, Lonardi S, Zagonel V, Salvatore L, et al. Initial therapy with FOLFOXIRI and bevacizumab for metastatic colorectal cancer. New Engl J Med. 2014;371:1609–18. 25337750 10.1056/NEJMoa1403108

[3] Mármol I, Sánchez-de-Diego C, Pradilla Dieste A, Cerrada E, Rodriguez Yoldi MJ. Colorectal carcinoma: a general overview and future perspectives in colorectal cancer. Int J Mol Sci. 2017;18:197. 28106826 10.3390/ijms18010197 PMC5297828

[4] Sinicrope FA, Shi Q, Smyrk TC, Thibodeau SN, Dienstmann R, Guinney J, et al. Molecular markers identify subtypes of stage III colon cancer associated with patient outcomes. Gastroenterology. 2015;148:88–99. 25305506 10.1053/j.gastro.2014.09.041 PMC4274188

[5] Suzuki N, Hazama S, Nagasaka T, Tanioka H, Iwamoto Y, Negoro Y, et al. Multicenter phase II study of biweekly CAPIRI plus bevacizumab as second-line therapy in patients with metastatic colorectal cancer (JSWOG-C3 study). Int J Clin Oncol. 2019;24:1223–30. 31144145 10.1007/s10147-019-01473-3 PMC6736909

[6] Battaglin F, Naseem M, Lenz HJ, Salem ME. Microsatellite instability in colorectal cancer: overview of its clinical significance and novel perspectives. Clin Adv Hematol Oncol. 2018;16:735–45. 30543589 PMC7493692

[7] Cervantes A, Adam R, Roselló S, Arnold D, Normanno N, Taïeb J, et al. Metastatic colorectal cancer: ESMO Clinical Practice Guideline for diagnosis, treatment and follow-up. Ann Oncol. 2023;34:10–32. 36307056 10.1016/j.annonc.2022.10.003

[8] Le DT, Uram JN, Wang H, Bartlett BR, Kemberling H, Eyring AD, et al. PD-1 blockade in tumors with mismatch-repair deficiency. New Engl J Med. 2015;372:2509–20. 26028255 10.1056/NEJMoa1500596 PMC4481136

[9] Overman MJ, McDermott R, Leach JL, Lonardi S, Lenz HJ, Morse MA, et al. Nivolumab in patients with metastatic DNA mismatch repair-deficient or microsatellite instability-high colorectal cancer (CheckMate 142): an open-label, multicentre, phase 2 study. Lancet Oncol. 2017;18:1182–91. 28734759 10.1016/S1470-2045(17)30422-9 PMC6207072

[10] Ganesh K, Stadler ZK, Cercek A, Mendelsohn RB, Shia J, Segal NH, et al. Immunotherapy in colorectal cancer: rationale, challenges and potential. Nat Rev Gastroenterol Hepatol. 2019;16:361–75. 30886395 10.1038/s41575-019-0126-x PMC7295073

[11] Syed YY. Durvalumab: first global approval. Drugs. 2017;77:1369–76. 28643244 10.1007/s40265-017-0782-5 PMC5636860

[12] Chen EX, Jonker DJ, Loree JM, Kennecke HF, Berry SR, Couture F, et al. Effect of combined immune checkpoint inhibition vs best supportive care alone in patients with advanced colorectal cancer: the Canadian Cancer Trials Group CO.26 Study. JAMA Oncol. 2020;6:831–8. 32379280 10.1001/jamaoncol.2020.0910 PMC7206536

[13] Saeed A, Park R, Dai J, Al-Rajabi R, Kasi A, Baranda J, et al. Cabozantinib plus durvalumab in advanced gastroesophageal cancer and other gastrointestinal malignancies: Phase Ib CAMILLA trial results. Cell Rep Med. 2023;4:100916. 36702123 10.1016/j.xcrm.2023.100916 PMC9975105

[14] Blay J, White TD, Hoskin DW. The extracellular fluid of solid carcinomas contains immunosuppressive concentrations of adenosine. Cancer Res. 1997;57:2602–5. 9205063

[15] Ohta A. A metabolic immune checkpoint: adenosine in tumor microenvironment. Front Immunol. 2016;7:109. 27066002 10.3389/fimmu.2016.00109 PMC4809887

[16] Stagg J, Smyth MJ. Extracellular adenosine triphosphate and adenosine in cancer. Oncogene. 2010;29:5346–58. 20661219 10.1038/onc.2010.292

[17] Roh M, Wainwright DA, Wu JD, Wan Y, Zhang B. Targeting CD73 to augment cancer immunotherapy. Curr Opin Pharmacol. 2020;53:66–76. 32777746 10.1016/j.coph.2020.07.001 PMC7669683

[18] Vijayan D, Young A, Teng MWL, Smyth MJ. Targeting immunosuppressive adenosine in cancer. Nat Rev Cancer. 2017;17:709–24. 29059149 10.1038/nrc.2017.86

[19] Hay CM, Sult E, Huang Q, Mulgrew K, Fuhrmann SR, McGlinchey KA, et al. Targeting CD73 in the tumor microenvironment with MEDI9447. Oncoimmunology. 2016;5:e1208875. 27622077 10.1080/2162402X.2016.1208875 PMC5007986

[20] Inoue Y, Yoshimura K, Kurabe N, Kahyo T, Kawase A, Tanahashi M, et al. Prognostic impact of CD73 and A2A adenosine receptor expression in non-small-cell lung cancer. Oncotarget. 2017;8:8738–51. 28060732 10.18632/oncotarget.14434 PMC5352437

[21] Messaoudi N, Cousineau I, Arslanian E, Henault D, Stephen D, Vandenbroucke-Menu F, et al. Prognostic value of CD73 expression in resected colorectal cancer liver metastasis. Oncoimmunology. 2020;9:1746138. 32363113 10.1080/2162402X.2020.1746138 PMC7185220

[22] Wu XR, He XS, Chen YF, Yuan RX, Zeng Y, Lian L, et al. High expression of CD73 as a poor prognostic biomarker in human colorectal cancer. J Surg Oncol. 2012;106:130–7. 22287455 10.1002/jso.23056

[23] Bendell J, LoRusso P, Overman M, Noonan AM, Kim DW, Strickler JH, et al. First-in-human study of oleclumab, a potent, selective anti-CD73 monoclonal antibody, alone or in combination with durvalumab in patients with advanced solid tumors. Cancer Immunol Immunother. 2023;72:2443–58. 37016126 10.1007/s00262-023-03430-6 PMC10264501

[24] Geoghegan JC, Diedrich G, Lu X, Rosenthal K, Sachsenmeier KF, Wu H, et al. Inhibition of CD73 AMP hydrolysis by a therapeutic antibody with a dual, non-competitive mechanism of action. MAbs. 2016;8:454–67. 26854859 10.1080/19420862.2016.1143182 PMC5037986

[25] Herbst RS, Majem M, Barlesi F, Carcereny E, Chu Q, Monnet I, et al. COAST: an open-label, phase II, multidrug platform study of durvalumab alone or in combination with oleclumab or monalizumab in patients with unresectable, stage III non-small-cell lung cancer. J Clin Oncol. 2022;40:3383–93. 35452273 10.1200/JCO.22.00227

[26] Martins I, Tesniere A, Kepp O, Michaud M, Schlemmer F, Senovilla L, et al. Chemotherapy induces ATP release from tumor cells. Cell Cycle. 2009;8:3723–8. 19855167 10.4161/cc.8.22.10026

[27] Eisenhauer EA, Therasse P, Bogaerts J, Schwartz LH, Sargent D, Ford R, et al. New response evaluation criteria in solid tumours: revised RECIST guideline (version 1.1). Eur J Cancer. 2009;45:228–47. 19097774 10.1016/j.ejca.2008.10.026

[28] Schulz KF, Altman DG, Moher D. CONSORT 2010 statement: updated guidelines for reporting parallel group randomized trials. Ann Intern Med. 2010;152:726–32. 20335313 10.7326/0003-4819-152-11-201006010-00232

[29] Klein JP, Logan B, Harhoff M, Andersen PK. Analyzing survival curves at a fixed point in time. Stat Med. 2007;26:4505–19. 17348080 10.1002/sim.2864

[30] Bendell JC, Powderly JD, Lieu CH, Eckhardt SG, Hurwitz H, Hochster HS. et al. Safety and efficacy of MPDL3280A (anti-PDL1) in combination with bevacizumab (bev) and/or FOLFOX in patients (pts) with metastatic colorectal cancer (mCRC). J Clin Oncol. 2015;33:704 10.1200/jco.2015.33.3_suppl.704

[31] Shahda S, Noonan AM, Bekaii-Saab TS, O’Neil BH, Sehdev A, Shaib WL. et al. A phase II tudy of pembrolizumab in combination with mFOLFOX6 for patients with advanced colorectal cancer. J Clin Oncol. 2017;35:3541. 10.1200/JCO.2017.35.15_suppl.3541

[32] El-Khoueiry AB, Fakih M, Gordon MS, Tsimberidou AM, Bullock AJ, Wilky BA. et al. Results from a phase 1a/1b study of botensilimab (BOT), a novel innate/adaptive immune activator, plus balstilimab (BAL; anti-PD-1 antibody) in metastatic heavily pretreated microsatellite stable colorectal cancer (MSS CRC). J Clin Oncol. 2023;41:LBA8. 10.1200/JCO.2023.41.4_suppl.LBA8

