# Supplementary material for: COLUMBIA-1: a randomised study of durvalumab plus oleclumab in combination with chemotherapy and bevacizumab in metastatic microsatellite-stable colorectal cancer
Source: Br J Cancer. 2024 Jul 25;131(6):1005–13. doi: 10.1038/s41416-024-02796-3 (PMC11405658; doi:10.1038/s41416-024-02796-3)
Supplement: Supplementary file 1 — Supplementary [file 41416_2024_2796_MOESM1_ESM.docx]

**SUPPLEMENTARY MATERIALS**

**Supplementary tables**

**Table S1.** Inclusion and exclusion criteria.

| **Inclusion criteria** |
| --- |
| **Informed consent** |
| Written informed consent and any locally required authorisation (e.g., HIPAA in the USA) obtained from the patient/legal representative prior to performing any protocol-related procedures, including screening evaluations. |
| **Age** |
| Age ≥18 years at the time of screening. |
| **Type of patient and disease characteristics** |
| ECOG performance status of 0 or 1. |
| Patients must have histological documentation of advanced or metastatic CRC and:   - A documented mutation test during screening and confirmed tumour locations from disease assessment for enrolment. - Patients must NOT have defective DNA MSI as documented by testing. Testing may be performed locally, and prior documentation of this testing is acceptable in lieu of repeating the test. Defective DNA mismatch repair is defined by either:   - High-frequency MSI with changes detected in ≥2 panels of microsatellite markers (BAT-25, BAT-26, NR-21, NR-24, or MONO-27), or   - Immunohistochemical analysis demonstrating absence of protein expression of any one or more of the following proteins: MLH1, MSH2, MSH6, or PMS2. - Patients must not have received any prior systemic therapy for recurrent/metastatic disease (prior adjuvant chemotherapy or chemoradiotherapy is acceptable so long as progression was not within <6 months of completing the adjuvant regimen). |
| Patients must have at least one lesion that is measurable by RECIST v1.1 [Eisenhauer et al, 2009].   - A previously irradiated lesion can be considered a target lesion if the lesion is well defined, measurable per RECIST, and has clearly progressed during or after the most recent therapy. - Up to 20 patients per arm at select centres undergoing pre-treatment and on treatment tumour biopsy must have a non-target, non-lymph node lesion that can be biopsied at acceptable risk as judged by the investigator or a target, non-lymph node lesion that can be biopsied and is ≥2 cm in longest diameter. Sites must confirm the nature of the tumour material (fresh vs archived FFPE) before treatment assignment. |
| Patients must have adequate organ function, as determined by:   - Haematological function (cannot be met with blood transfusions or growth factor support within <2 weeks of scheduled first dose of study treatment):   - Absolute neutrophil count ≥1.5 × 10^9^/L.   - Platelet count ≥100 × 10^9^/L.   - Haemoglobin ≥9.0 g/dL. - Renal function:   - Calculated creatinine clearance by the modification of diet in renal disease equation [Levey et al, 2006] or 24 h urine creatinine clearance >40 mL/min.   - Urinalysis ≤1+ protein. Patients discovered to have ≥2+ proteinuria at baseline must undergo a 24 h urine collection that must demonstrate <1 g of protein/24 h or have a UPC ratio <1.0. - Hepatic function:   - Total bilirubin ≤1.5 × ULN if no demonstrable liver metastases or ≤3 ULN in the presence of documented Gilbert’s syndrome or liver metastases.   - ALT and AST ≤2.5 × ULN if no demonstrable liver metastases or ≤5 × ULN in the presence of liver metastasis. - Coagulation function:   - INR <1.5 × ULN, with the exception of patients on systemic anticoagulation   - Partial thromboplastin time (or activated partial thromboplastin time) <1.5 × ULN. |
| Patients with medical conditions requiring systemic anticoagulation (e.g., atrial fibrillation) are eligible provided that both of the following criteria are met:   - The patient has an in-range INR (usually 2–3) on a stable dose of oral anticoagulant or be on a stable dose of low molecular weight heparin. - The patient has no active bleeding or pathological condition that carries a high risk of bleeding (e.g., tumour involving major vessels or known varices).   **NOTE:** Patients receiving antiplatelet agents including daily prophylactic aspirin (≤325 mg/day) are permitted to enrol. In addition, patients with a venous thrombosis are permitted to enrol, including patients on newer oral anticoagulants (e.g., apixaban, rivaroxaban, dabigatran, etc.), provided they are clinically stable, asymptomatic, and adequately treated with anticoagulation in the opinion of the investigator, for ≥3 months prior to the scheduled first dose of study treatment. |
| **Weight** |
| Body weight >35 kg. |
| **Lifestyle/reproduction** |
| Females of childbearing potential who are sexually active with a non-sterilised male partner must have used at least one highly effective method of contraception from screening through 180 days after the final dose of investigational product. It is strongly recommended for the male partner of a female patient to also use male condom plus spermicide (except in countries where spermicides are not approved) throughout this period. Cessation of contraception after this point should be discussed with a responsible physician. Periodic abstinence, the rhythm method, and the withdrawal method are not acceptable methods of contraception. In addition, female patients must refrain from egg cell donation and breastfeeding while on study and for 180 days after the final dose of investigational product. |
| Non-sterilised male patients who are sexually active with a female partner of childbearing potential must use a male condom with spermicide (except in countries where spermicides are not approved) from screening through 180 days after receipt of the final dose of investigational product. It is strongly recommended for the female partner of a male patient to also use a highly effective method of contraception throughout this period. In addition, male patients must refrain from sperm donation while on study and for 180 days after the final dose of investigational product. |
| **Exclusion criteria** |
| **Medical conditions** |
| Any condition that, in the opinion of the investigator, would interfere with evaluation of the investigational product or interpretation of patient safety or study results. |
| History of allogeneic organ transplantation. |
| Active or prior documented autoimmune disorders within the past 5 years prior to the scheduled first dose of study treatment. The following are exceptions to this criterion:   - Patients with vitiligo or alopecia. - Patients with hypothyroidism (e.g., following Hashimoto syndrome) stable on hormone replacement. - Any chronic skin condition that does not require systemic therapy. - Patients with coeliac disease controlled by diet alone. |
| History of venous thrombosis within the past 3 months prior to the scheduled first dose of study treatment. |
| Cardiovascular criteria:   - Presence of acute coronary syndrome including myocardial infarction or unstable angina pectoris, other arterial thrombotic event including cerebrovascular accident or transient ischemic attack or stroke within the past 6 months prior to the scheduled first dose of study treatment. - New York Heart Association Class II or greater congestive heart failure, serious cardiac arrhythmia requiring medication, or uncontrolled hypertension (≥160 mmHg systolic and/or ≥100 mmHg diastolic, despite appropriate antihypertensive medication). - History of hypertensive crisis/hypertensive encephalopathy within the past 6 months prior to the scheduled first dose of study treatment. |
| Mean QT interval corrected for heart rate using Fridericia’s formula (QTcF) ≥470 ms calculated from 3 ECGs obtained within a 5 min period at least 1 min apart. |
| No significant history of bleeding events or gastrointestinal perforation:   - History of significant bleeding episodes (e.g., haemoptysis, upper or lower gastrointestinal bleeding) within the past 6 months unless the source of bleeding has been resected. - History of gastrointestinal perforation within the past 12 months prior to the scheduled first dose of study treatment. |
| Uncontrolled intercurrent illness, including but not limited to, ongoing or active infection, interstitial lung disease, serious chronic gastrointestinal conditions associated with diarrhoea, or psychiatric illness/social situations that would limit compliance with study requirement, substantially increase risk of incurring AEs, or compromise the ability of the patient to give written informed consent. |
| History of another primary malignancy except for:   - Malignancy treated with curative intent and with no known active disease ≥5 years prior to the scheduled first dose of study treatment and of low potential risk for recurrence. - Adequately treated non-melanoma skin cancer or lentigo maligna without evidence of disease. - Adequately treated carcinoma in situ without evidence of disease. |
| History of active primary immunodeficiency. |
| Active infection including tuberculosis (clinical evaluation that includes clinical history, physical examination and radiographic findings, and tuberculosis testing in line with local practice), hepatitis B (known positive HBV surface antigen [hBsAg] result), hepatitis C, or HIV (positive HIV 1/2 antibodies).  **NOTE:** Patients with a past or resolved HBV infection (defined as the presence of hepatitis B core antibody [anti-HBc] and absence of hBsAg) are eligible. Patients positive for HCV antibody are eligible only if polymerase chain reaction is negative for HCV RNA. |
| Known allergy or hypersensitivity to any of the study drugs or any of the study drug excipients. |
| Any unresolved toxicity NCI CTCAE Grade >1 from previous anticancer therapy with the exception of alopecia, vitiligo, and the laboratory values defined in the inclusion criteria. |
| History of leptomeningeal disease or cord compression. |
| Untreated CNS metastases identified either on the baseline brain imaging obtained during the screening period or identified prior to signing the informed consent form.  **NOTE:** Patients whose brain metastases have been treated may participate provided they show radiographic stability (defined as two brain images, both of which are obtained after treatment to the brain metastases. These imaging scans should both be obtained at least 4 weeks apart and show no evidence of intracranial progression). In addition, any neurological symptoms that developed either as a result of the brain metastases or their treatment must have resolved or be stable, without the use of steroids for at least 14 days prior to the scheduled first dose of study treatment. Brain metastases will not be recorded as RECIST target lesions at baseline. |
| Lack of physical integrity of the upper gastrointestinal tract, malabsorption syndrome, or inability to take oral medication. |
| Known DPD deficiency. Testing for DPD deficiency must be performed where required by local regulations, using a validated method that is approved by local health authorities. |
| **Prior/concomitant therapy** |
| Any concurrent chemotherapy, investigational product, biological, or hormonal therapy for cancer treatment. Concurrent use of hormonal therapy for non-cancer-related conditions (e.g., hormone replacement therapy) is acceptable. |
| Radiotherapy treatment to more than 30% of the bone marrow or with a wide field of radiation within 4 weeks prior to the scheduled first dose of study treatment. |
| Prior receipt of any immune-mediated therapy including, but not limited to, other anti-CTLA-4, anti-PD-1, anti-PD-L1 antibodies, and agents targeting CD73, CD39, or adenosine receptors, excluding therapeutic anticancer vaccines. |
| Prior receipt of anti-angiogenics, including, but not limited to, VEGF or VEGF receptor inhibitors. |
| Receipt of live attenuated vaccine within 30 days prior to the scheduled first dose of study treatment. |
| Major surgical procedure, open biopsy, or significant traumatic injury (all as defined by the investigator) within 28 days prior to the scheduled first dose of study treatment, or anticipation of the need for major surgical procedure during the course of the study.  **NOTE:** Local surgery of isolated lesions for palliative intent is acceptable. |
| Current or prior use of immunosuppressive medication within 14 days prior to the scheduled first dose of study treatment. The following are exceptions to this criterion:   - Intranasal, inhaled, topical steroids, or local steroid injections (e.g., intra-articular injection). - Steroids as premedication for hypersensitivity reactions (e.g., CT scan premedication or chemotherapy premedication per institutional practice). |
| **Prior/concurrent clinical study experience** |
| Participation in another clinical study with an investigational product administered within 28 days prior to the scheduled first dose of study treatment. |
| Concurrent enrolment in another clinical study unless it is an observational (noninterventional) clinical study or during the follow-up period of an interventional study. |
| **Other exclusions** |
| Female patients who are pregnant, breastfeeding, or intend to become pregnant during their participation in the study. |
| Involvement in the planning and/or conduct of the study (applies to both sponsor staff and/or staff at the study site). |
| **Genetic research study (optional)** |
| Exclusion criteria for participation in the optional genetics research component of the study include:   - Non-leukocyte-depleted whole blood transfusion within 120 days of genetic sample collection. |

Eisenhauer EA, Therasse P, Bogaerts J, et al. New response evaluation criteria in solid tumours: Revised RECIST guideline (version 1.1). Eur J Cancer 2009; 45:228–247.

Levey AS, Coresh J, Greene T, et al. Using Standardized Serum Creatinine Values in the Modification of Diet in Renal Disease Study Equation for Estimating Glomerular Filtration Rate. Ann Intern Med 2006; 145:247–254.

AE, adverse event; ALT, alanine transaminase; AST, aspartate transaminase; CNS, central nervous system; CRC, colorectal cancer; CT, computed tomography; CTCAE, Common Terminology Criteria for Adverse Events; CTLA-4, cytotoxic T-lymphocyte-associated antigen 4; DPD, dihydropyrimidine dehydrogenase; ECG, electrocardiogram; ECOG, Eastern Cooperative Oncology Group; FFPE, formalin-fixed paraffin embedded; HBV, hepatitis B virus; HCV, hepatitis C virus; HIPAA, Health Insurance Portability and Accountability Act; HIV, human immunodeficiency virus; INR, international normalised ratio; MSI, microsatellite instability; NCI, National Cancer Institute; PD-1, programmed cell death-1; PD-L1, programmed death-ligand 1; RECIST, response evaluation criteria in solid tumours; ULN, upper limit of normal; UPC, urine protein:creatinine; USA, United States of America; VEGF, vascular endothelial growth factor.

**Table S2.** CONSORT statement checklist for reports of cohort studies.

|  |  | **Reporting Item** | **Page Number** |
| --- | --- | --- | --- |
| **Title and Abstract** | | | |
| Title | [#1a](https://www.goodreports.org/reporting-checklists/consort/info/#1a) | Identification as a randomized trial in the title. | 1 |
| Abstract | [#1b](https://www.goodreports.org/reporting-checklists/consort/info/#1b) | Structured summary of trial design, methods, results, and conclusions | 3 |
| **Introduction** | | | |
| Background and objectives | [#2a](https://www.goodreports.org/reporting-checklists/consort/info/#2a) | Scientific background and explanation of rationale | 4 |
| Background and objectives | [#2b](https://www.goodreports.org/reporting-checklists/consort/info/#2b) | Specific objectives or hypothesis | 5 |
| **Methods** | | | |
| Trial design | [#3a](https://www.goodreports.org/reporting-checklists/consort/info/#3a) | Description of trial design (such as parallel, factorial) including allocation ratio. | 6 |
| Trial design | [#3b](https://www.goodreports.org/reporting-checklists/consort/info/#3b) | Important changes to methods after trial commencement (such as eligibility criteria), with reasons | NA |
| Participants | [#4a](https://www.goodreports.org/reporting-checklists/consort/info/#4a) | Eligibility criteria for participants | 5, 30 |
| Participants | [#4b](https://www.goodreports.org/reporting-checklists/consort/info/#4b) | Settings and locations where the data were collected | 6 |
| Interventions | [#5](https://www.goodreports.org/reporting-checklists/consort/info/#5) | The experimental and control interventions for each group with sufficient details to allow replication, including how and when they were actually administered | 6 |
| Outcomes | [#6a](https://www.goodreports.org/reporting-checklists/consort/info/#6a) | Completely defined prespecified primary and secondary outcome measures, including how and when they were assessed | 6 |
| Outcomes | [#6b](https://www.goodreports.org/reporting-checklists/consort/info/#6b) | Any changes to trial outcomes after the trial commenced, with reasons | NA |
| Sample size | [#7a](https://www.goodreports.org/reporting-checklists/consort/info/#7a) | How sample size was determined. | 7 |
| Sample size | [#7b](https://www.goodreports.org/reporting-checklists/consort/info/#7b) | When applicable, explanation of any interim analyses and stopping guidelines | 8 |
| Randomisation - Sequence generation | [#8a](https://www.goodreports.org/reporting-checklists/consort/info/#8a) | Method used to generate the random allocation sequence. | 8 |
| Randomisation - Sequence generation | [#8b](https://www.goodreports.org/reporting-checklists/consort/info/#8b) | Type of randomization; details of any restriction (such as blocking and block size) | 8 |
| Randomisation - Allocation concealment mechanism | [#9](https://www.goodreports.org/reporting-checklists/consort/info/#9) | Mechanism used to implement the random allocation sequence (such as sequentially numbered containers), describing any steps taken to conceal the sequence until interventions were assigned | 8 |
| Randomisation - Implementation | [#10](https://www.goodreports.org/reporting-checklists/consort/info/#10) | Who generated the allocation sequence, who enrolled participants, and who assigned participants to interventions | 8 |
| Blinding | [#11a](https://www.goodreports.org/reporting-checklists/consort/info/#11a) | If done, who was blinded after assignment to interventions (for example, participants, care providers, those assessing outcomes) and how | NA |
| Blinding | [#11b](https://www.goodreports.org/reporting-checklists/consort/info/#11b) | If relevant, description of the similarity of interventions | NA |
| Statistical methods | [#12a](https://www.goodreports.org/reporting-checklists/consort/info/#12a) | Statistical methods used to compare groups for primary and secondary outcomes | 8 |
| Statistical methods | [#12b](https://www.goodreports.org/reporting-checklists/consort/info/#12b) | Methods for additional analyses, such as subgroup analyses and adjusted analyses | 8 |
| **Results** | | | |
| Participant flow diagram (strongly recommended) | [#13a](https://www.goodreports.org/reporting-checklists/consort/info/#13a) | For each group, the numbers of participants who were randomly assigned, received intended treatment, and were analysed for the primary outcome | 8 |
| Participant flow | [#13b](https://www.goodreports.org/reporting-checklists/consort/info/#13b) | For each group, losses and exclusions after randomisation, together with reason | NA |
| Recruitment | [#14a](https://www.goodreports.org/reporting-checklists/consort/info/#14a) | Dates defining the periods of recruitment and follow-up | 6 |
| Recruitment | [#14b](https://www.goodreports.org/reporting-checklists/consort/info/#14b) | Why the trial ended or was stopped | NA |
| Baseline data | [#15](https://www.goodreports.org/reporting-checklists/consort/info/#15) | A table showing baseline demographic and clinical characteristics for each group | 19 |
| Numbers analysed | [#16](https://www.goodreports.org/reporting-checklists/consort/info/#16) | For each group, number of participants (denominator) included in each analysis and whether the analysis was by original assigned groups | 8–10, 19–27 |
| Outcomes and estimation | [#17a](https://www.goodreports.org/reporting-checklists/consort/info/#17a) | For each primary and secondary outcome, results for each group, and the estimated effect size and its precision (such as 95% confidence interval) | 9–10 |
| Outcomes and estimation | [#17b](https://www.goodreports.org/reporting-checklists/consort/info/#17b) | For binary outcomes, presentation of both absolute and relative effect sizes is recommended | NA |
| Ancillary analyses | [#18](https://www.goodreports.org/reporting-checklists/consort/info/#18) | Results of any other analyses performed, including subgroup analyses and adjusted analyses, distinguishing pre-specified from exploratory | 10 |
| Harms | [#19](https://www.goodreports.org/reporting-checklists/consort/info/#19) | All important harms or unintended effects in each group (For specific guidance see CONSORT for harms) | 9, 22–25 |
| **Discussion** | | | |
| Limitations | [#20](https://www.goodreports.org/reporting-checklists/consort/info/#20) | Trial limitations, addressing sources of potential bias, imprecision, and, if relevant, multiplicity of analyses | 12 |
| Generalisability | [#21](https://www.goodreports.org/reporting-checklists/consort/info/#21) | Generalisability (external validity, applicability) of the trial findings | 11–12 |
| Interpretation | [#22](https://www.goodreports.org/reporting-checklists/consort/info/#22) | Interpretation consistent with results, balancing benefits and harms, and considering other relevant evidence | 12 |
| Registration | [#23](https://www.goodreports.org/reporting-checklists/consort/info/#23) | Registration number and name of trial registry | 5 |
| **Other information** | | | |
| Interpretation | [#22](https://www.goodreports.org/reporting-checklists/consort/info/#22) | Interpretation consistent with results, balancing benefits and harms, and considering other relevant evidence | 12 |
| Registration | [#23](https://www.goodreports.org/reporting-checklists/consort/info/#23) | Registration number and name of trial registry | 5 |
| Protocol | [#24](https://www.goodreports.org/reporting-checklists/consort/info/#24) | Where the full trial protocol can be accessed, if available | 13 |
| Funding | [#25](https://www.goodreports.org/reporting-checklists/consort/info/#25) | Sources of funding and other support (such as supply of drugs), role of funders | 15 |

**Table S3.** Treatment exposure.

|  | **Part 1** | **Part 2** | | |
| --- | --- | --- | --- | --- |
| **Median duration of exposure** | **FOLFOX + bevacizumab + durvalumab + oleclumab (n=7)** | **Total (N=52)** | **Control arm: FOLFOX + bevacizumab (n=26)** | **Experimental arm: FOLFOX + bevacizumab + durvalumab + oleclumab (n=26)** |
| **Durvalumab** | | | | |
| Weeks (range) | 44.0 (32–130) | 34.3 (4–122) | N/A | 34.3 (4–122) |
| Cycles (range) | 11.0 (7–29) | 8.5 (1–28) | N/A | 8.5 (1–28) |
| **Oleclumab** | | | | |
| Weeks (range) | 44.0 (32–130) | 34.3 (2–122) | N/A | 34.3 (2–122) |
| Cycles (range) | 13.0 (9–31) | 10.5 (1–30) | N/A | 10.5 (1–30) |
| **Folinic acid** | | | | |
| Weeks (range) | 56.0 (41–128) | 32.5 (2–120) | 32.7 (2–101) | 31.3 (8–120) |
| Cycles (range) | 27.0 (19–57) | 15.5 (1–56) | 15.5 (1–41) | 15.5 (4–56) |
| **Oxaliplatin** | | | | |
| Weeks (range) | 25.0 (6–33) | 22.2 (2–64) | 23.2 (2–64) | 22.0 (4–46) |
| Cycles (range) | 12.0 (3–15) | 10.5 (1–27) | 11.0 (1–27) | 10.5 (2–18) |
| **5-Fluorouracil** | | | | |
| Weeks (range) | 56.3 (41–128) | 33.8 (2–120) | 33.0 (2–101) | 36.8 (8–120) |
| Cycles (range) | 27.0 (19–57) | 16.0 (1–55) | 15.5 (1–41) | 17.0 (4–55) |
| **Bevacizumab** | | | | |
| Weeks (range) | 43.9 (18–128) | 32.7 (2–120) | 32.7 (2–101) | 33.3 (2–120) |
| Cycles (range) | 21.0 (6–57) | 15.5 (1–56) | 15.5 (1–41) | 16.0 (1–56) |

Duration of exposure (weeks) for patients on treatment was defined as: The last dose date plus 14 days (for FOLFOX and bevacizumab) or 28 days (for durvalumab) minus first dose date; or for oleclumab, the last dose date plus 14 days minus first dose date (for patients on treatment prior to Cycle 3 Day 1) or last dose date plus 28 days minus first dose date (for those on treatment post Cycle 3 Day 1). For patients who discontinued treatment: a minimum date of death plus 1 day, data cut-off date plus 1 day, last dose date plus 14 days (for FOLFOX and bevacizumab) or 28 days (for durvalumab) minus first dose date; or for oleclumab, a minimum of date of death plus 1 day, data cut-off date plus 1 day, last dose date plus 14 days for patients who discontinued treatment prior to Cycle 3 Day 1 or 28 days for those who discontinued treatment after Cycle 3 Day 1.
Duration of exposure (cycles) was defined as: the number of cycles in which at least one portion of investigational agent was administered.

FOLFOX, folinic acid, 5-fluorouracil, and oxaliplatin; N/A, not applicable.

**Table S4.** TRAEs occurring in ≥10% of patients in Part 2 (total).

|  | **Part 1** | **Part 2** | | |
| --- | --- | --- | --- | --- |
| **TRAEs, n (%)** | **FOLFOX + bevacizumab + durvalumab + oleclumab (n=7)** | **Total (N=52)** | **Control arm: FOLFOX + bevacizumab (n=26)** | **Experimental arm: FOLFOX + bevacizumab + durvalumab + oleclumab (n=26)** |
| **Durvalumab** | | | | |
| Fatigue | 1 (14.3) | 6 (11.5) | N/A | 6 (23.1) |
| **Oleclumab** | | | | |
| Fatigue | 1 (14.3) | 6 (11.5) | N/A | 6 (23.1) |
| **Folinic acid** | | | | |
| Nausea | 0 | 15 (28.8) | 8 (30.8) | 7 (26.9) |
| Diarrhoea | 0 | 12 (23.1) | 5 (19.2) | 7 (26.9) |
| Fatigue | 1 (14.3) | 11 (21.2) | 5 (19.2) | 6 (23.1) |
| Dysgeusia | 0 | 7 (13.5) | 5 (19.2) | 2 (7.7) |
| **Oxaliplatin** | | | | |
| Peripheral sensory neuropathy | 5 (71.4) | 21 (40.4) | 11 (42.3) | 10 (38.5) |
| Nausea | 1 (14.3) | 20 (38.5) | 13 (50.0) | 7 (26.9) |
| Fatigue | 5 (71.4) | 16 (30.8) | 10 (38.5) | 6 (23.1) |
| Diarrhoea | 3 (42.9) | 14 (26.9) | 7 (26.9) | 7 (26.9) |
| Paraesthesia | 5 (71.4) | 12 (23.1) | 7 (26.9) | 5 (19.2) |
| Dysgeusia | 2 (28.6) | 10 (19.2) | 7 (26.9) | 3 (11.5) |
| Stomatitis | 1 (14.3) | 9 (17.3) | 3 (11.5) | 6 (23.1) |
| Temperature intolerance | 1 (14.3) | 9 (17.3) | 4 (15.4) | 5 (19.2) |
| Neuropathy peripheral | 0 | 8 (15.4) | 3 (11.5) | 5 (19.2) |
| Neutrophil count decreased | 4 (57.1) | 7 (13.5) | 3 (11.5) | 4 (15.4) |
| Platelet count decreased | 1 (14.3) | 7 (13.5) | 4 (15.4) | 3 (11.5) |
| Decreased appetite | 0 | 6 (11.5) | 4 (15.4) | 2 (7.7) |
| **5-Fluorouracil** | | | | |
| Nausea | 2 (28.6) | 22 (42.3) | 13 (50.0) | 9 (34.6) |
| Diarrhoea | 4 (57.1) | 19 (36.5) | 8 (30.8) | 11 (42.3) |
| Fatigue | 5 (71.4) | 16 (30.8) | 10 (38.5) | 6 (23.1) |
| Dysgeusia | 2 (28.6) | 8 (15.4) | 7 (26.9) | 1 (3.8) |
| Neutrophil count decreased | 4 (57.1) | 8 (15.4) | 4 (15.4) | 4 (15.4) |
| Stomatitis | 3 (42.9) | 8 (15.4) | 3 (11.5) | 5 (19.2) |
| Vomiting | 0 | 8 (15.4) | 3 (11.5) | 5 (19.2) |
| Platelet count decreased | 1 (14.3) | 6 (11.5) | 4 (15.4) | 2 (7.7) |
| Decreased appetite | 1 (14.3) | 6 (11.5) | 4 (15.4) | 2 (7.7) |
| **Bevacizumab** | | | | |
| Nausea | 0 | 15 (28.8) | 9 (34.6) | 6 (23.1) |
| Fatigue | 1 (14.3) | 13 (25.0) | 6 (23.1) | 7 (26.9) |
| Diarrhoea | 1 (14.3) | 12 (23.1) | 5 (19.2) | 7 (26.9) |
| Epistaxis | 0 | 7 (13.5) | 4 (15.4) | 3 (11.5) |
| Dysgeusia | 0 | 6 (11.5) | 5 (19.2) | 1 (3.8) |

FOLFOX, folinic acid, 5-fluorouracil, and oxaliplatin; NA, not applicable; TRAE, treatment-related adverse events.

**Table S5.** Efficacy outcomes by tumoural CD73 expression status.

|  | **CD73 low** | | **CD73 high** | |
| --- | --- | --- | --- | --- |
|  | **Control arm FOLFOX + bevacizumab (n=10)** | **Experimental arm FOLFOX + bevacizumab + durvalumab + oleclumab (n=12)** | **Control arm: FOLFOX + bevacizumab (n=13*)** | **Experimental arm: FOLFOX + bevacizumab + durvalumab + oleclumab (n=11)** |
| **ORR, % (95% CI)** | 60.0 (26.2–87.8) | 58.3 (27.7–84.8) | 35.7 (12.8–64.9) | 72.7 (39–94) |
| **Median PFS, months (95% CI)** | 8.3 (6.7–NE) | 7.4 (5.6–NE) | 11.2 (7.5–NE) | 11.3 (9.2–NE) |
| **Median OS, months (95% CI)** | 20.7 (12.5–NE) | 22.4 (10.6–NE) | NE | 19.1 (13.9–NE) |

*One patient in the control arm with high CD73 was not response-evaluable (total n=14).

CI, confidence interval; FOLFOX, folinic acid, 5-fluorouracil, and oxaliplatin; NE, not evaluable; ORR, objective response rate; OS, overall survival; PFS, progression-free survival.

**Supplementary figures**

**Figure S1.** COLUMBIA-1 (NCT04068610) study design


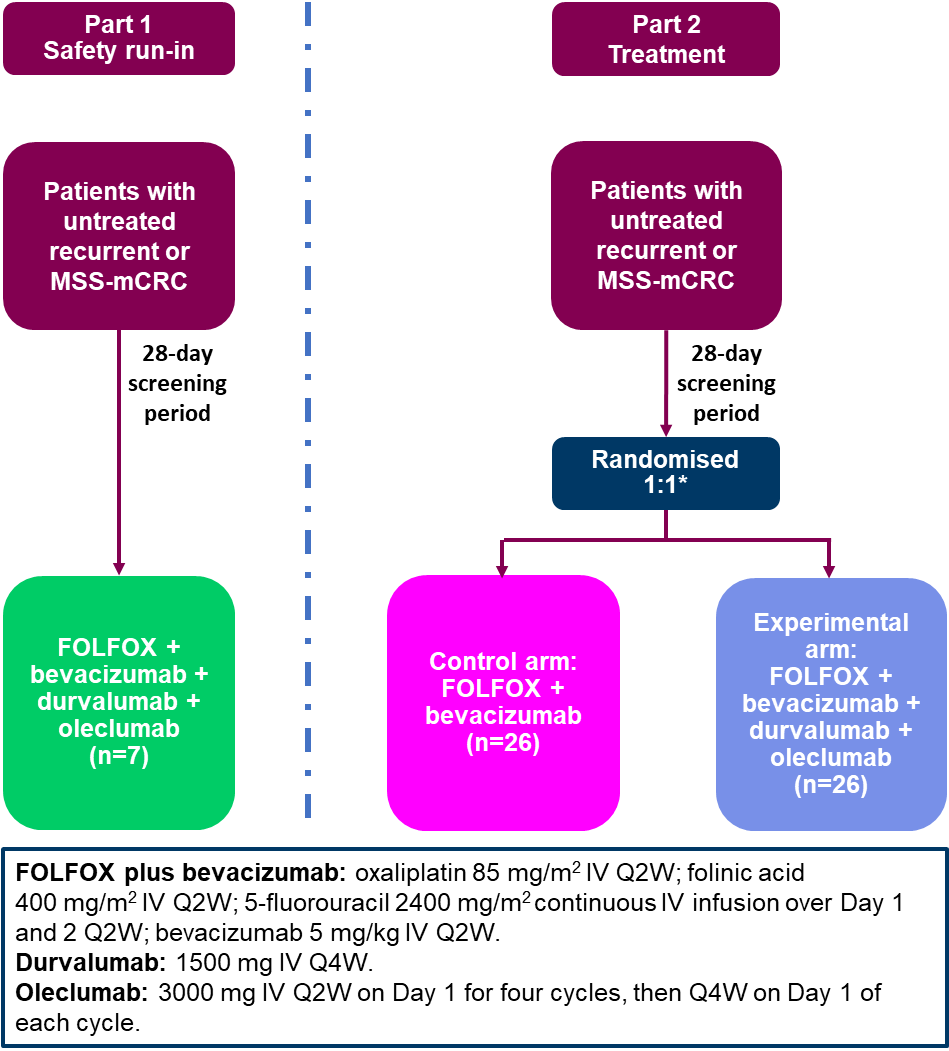


*Randomisation is stratified by tumour location (left-sided vs right-sided).

FOLFOX, folinic acid, 5-fluorouracil and oxaliplatin; IV, intravenous; mCRC, metastatic colorectal cancer; MSS, microsatellite stable; Q*x*W, every *x* weeks.

**Figure S2.** Best change in tumour size from baseline by tumoral CD73 expression status.


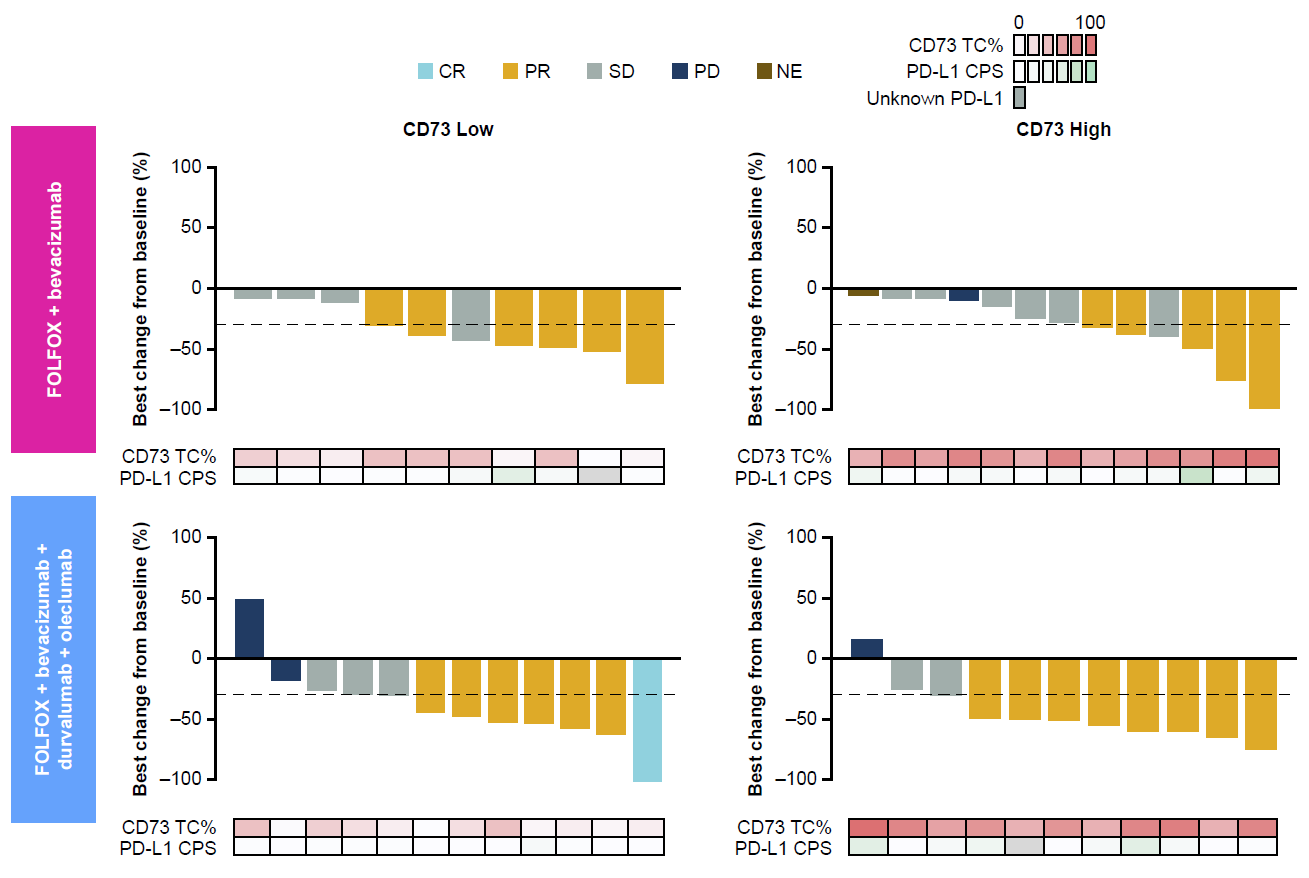


CD73, cluster of differentiation 73; CPS, combined positive score; CR, complete response; FOLFOX, folinic acid, 5-fluorouracil, and oxaliplatin; NE, not evaluable; PD, progressive disease; PD-L1, programmed cell death ligand-1; PR, partial response; SD, stable disease; TC%, percentage of tumour cells.

**Figure S3**. Survival data using the Kaplan–Meier methods to indicate PFS (A and B) and OS (C and D) by level of tumoral CD73 expression.


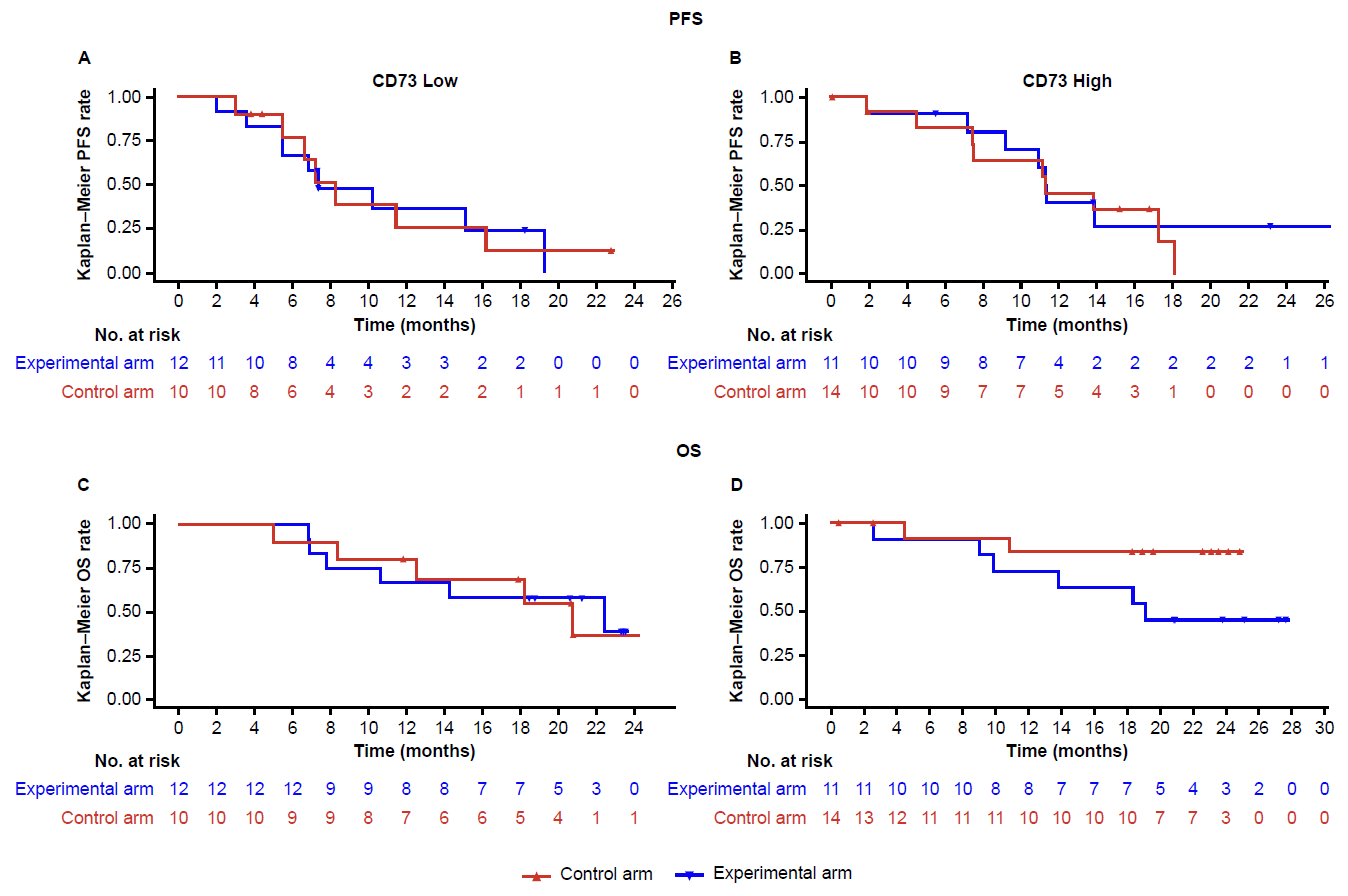


Control arm: FOLFOX + bevacizumab. Experimental arm: FOLFOX + bevacizumab + durvalumab + oleclumab.

FOLFOX, folinic acid, 5-fluorouracil, and oxaliplatin; OS, overall survival; PFS, progression-free survival.
